# Supplementary material for: Structural and functional brain changes in people with knee osteoarthritis: a scoping review
Source: PeerJ. 2023 Sep 7;11:e16003. doi: 10.7717/peerj.16003 (PMC10493091; doi:10.7717/peerj.16003)
Supplement: Supplemental Information 2 [file peerj-11-16003-s002.docx]

**Supplementary material.**

Search strategy in MEDLINE (PubMed)

((((((((((((osteoarthritis) OR (Osteoarthritides)) OR (Osteoarthrosis)) OR (Osteoarthroses)) OR (Arthrosis)) OR (Arthroses)) OR (OA))) AND ((((((((((((((((((((((((((Brain Mapping) OR (brain imaging)) OR (Magnetic resonance)) OR (MR imaging)) OR (MRI)) OR (fMRI)) OR (rs-fMRI)) OR (Resting state fMRI)) OR (Voxel-based Morphometry)) OR (VBM)) OR (Difussion tensor imaging)) OR (DTI)) OR (EEG)) OR (Electroencephalography)) OR (Electrocorticography)) OR (MEG)) OR (Magnetoencephalography)) OR (PET)) OR (Positron emission tomography)) OR (Surface Based Morphometry)) OR (Neuroimaging)) OR (Functional near-infrared spectroscopy)) OR (near-infrared spectroscopy)) OR (optical imaging system)) OR (optical topography)))) AND (((((((((((((((((((((Brain Cortical thickness) OR (Cerebral Blood Flow)) OR (Cerebral oxygenation)) OR (Brain morphology)) OR (Brain Morphometry)) OR (Brain volume)) OR (Neuroplasticity)) OR (Neuroelectric)) OR (Electrophysiological)) OR (Event-related potentials)) OR (ERP)) OR (Alpha waves)) OR (Beta waves)) OR (Gamma waves)) OR (Theta waves)) OR (Gray matter)) OR (Grey matter)) OR (White matter)) OR (Functional connectivity)) OR (Structural connectivity))))) NOT ((((((((STROKE) OR (Cancer)) OR (Neoplasms)) OR (Brain Injuries, Traumatic)) OR (Neurodegenerative Diseases)) OR (Alzheimer Disease)) OR (Parkinson Disease))))
